# *QuickStats:* Percentage* of Adults Aged ≥18 Years Who Daily Experienced Feelings of Anxiety (Feeling Worried, Nervous, or Anxious)^†^ or Depression,^§^ or Both, by Sex — National Health Interview Survey,^¶^ United States, 2019

**DOI:** 10.15585/mmwr.mm7032a5

**Published:** 2021-08-13

**Figure:**
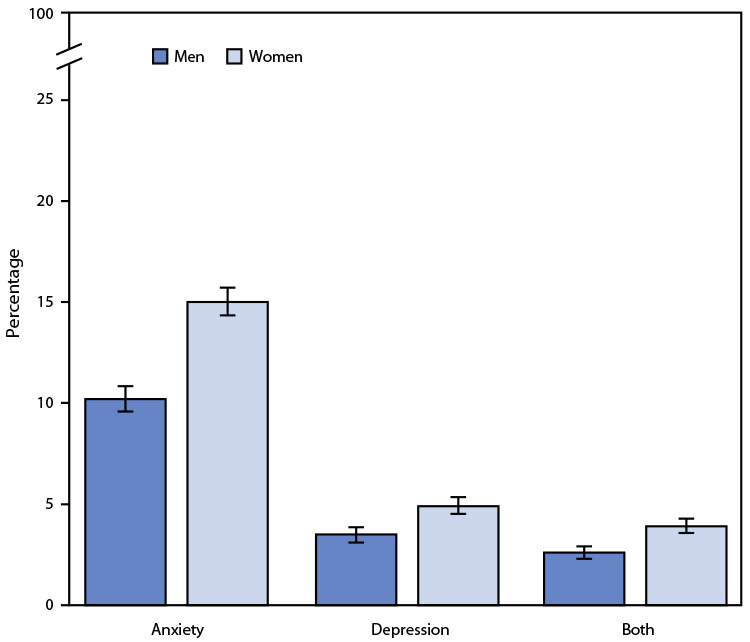
In 2019, women were more likely than men to feel worried, nervous, or anxious on a daily basis (15.0% versus 10.2%). Women were also more likely to feel depressed daily (4.9%) compared with men (3.5%). A higher percentage of women than men reported experiencing daily feelings of both anxiety and depression (3.9% versus 2.6%).

**Source:** National Center for Health Statistics, National Health Interview Survey, 2019. https://www.cdc.gov/nchs/nhis/index.htm

